# Spatial and spatio-temporal analysis of malaria cases in Zimbabwe

**DOI:** 10.1186/s40249-020-00764-6

**Published:** 2020-10-22

**Authors:** Isaiah Gwitira, Munashe Mukonoweshuro, Grace Mapako, Munyaradzi D. Shekede, Joconiah Chirenda, Joseph Mberikunashe

**Affiliations:** 1 Department of Geography Geospatial Sciences and Earth Observation, University of Zimbabwe, P. O. Box MP 167, Mount Pleasant, Harare Zimbabwe; 2 Department of Community Medicine, University of Zimbabwe, 3rd Floor New Health Sciences Building, College of Health Sciences, P O Box A178, Avondale, Harare Zimbabwe; 3 National Malaria Control Program, Ministry of Health and Child Care, 4th Floor, Kaguvi Building, Central Avenue (Between 4th and 5th Street), Harare, Zimbabwe

**Keywords:** Malaria, GIS, SaTscan, Spatial pattern, Spatial heterogeneity, Cluster analysis, Zimbabwe

## Abstract

**Background:**

Although effective treatment for malaria is now available, approximately half of the global population remain at risk of the disease particularly in developing countries. To design effective malaria control strategies there is need to understand the pattern of malaria heterogeneity in an area. Therefore, the main objective of this study was to explore the spatial and spatio-temporal pattern of malaria cases in Zimbabwe based on malaria data aggregated at district level from 2011 to 2016.

**Methods:**

Geographical information system (GIS) and spatial scan statistic were applied on passive malaria data collected from health facilities and aggregated at district level to detect existence of spatial clusters. The global Moran’s *I* test was used to infer the presence of spatial autocorrelation while the purely spatial retrospective analyses were performed to detect the spatial clusters of malaria cases with high rates based on the discrete Poisson model. Furthermore, space-time clusters with high rates were detected through the retrospective space-time analysis based on the discrete Poisson model.

**Results:**

Results showed that there is significant positive spatial autocorrelation in malaria cases in the study area. In addition, malaria exhibits spatial heterogeneity as evidenced by the existence of statistically significant (*P* < 0.05) spatial and space-time clusters of malaria in specific geographic regions. The detected primary clusters persisted in the eastern region of the study area over the six year study period while the temporal pattern of malaria reflected the seasonality of the disease where clusters were detected within particular months of the year.

**Conclusions:**

Geographic regions characterised by clusters of high rates were identified as malaria high risk areas. The results of this study could be useful in prioritizing resource allocation in high-risk areas for malaria control and elimination particularly in resource limited settings such as Zimbabwe. The results of this study are also useful to guide further investigation into the possible determinants of persistence of high clusters of malaria cases in particular geographic regions which is useful in reducing malaria burden in such areas.

## Background

Compared with other human diseases, malaria remains one of the most serious public health problem associated with high morbidity and mortality in most developing countries [1–3]. In 2018 alone, 228 million malaria cases and 405 000 deaths were recorded worldwide with the World Health Organisation (WHO) African Region contributing 93% of the cases and 94% of the deaths [4]. Although malaria has been successfully eradicated in high income and most middle income countries, the disease remains a major health problem and is a top killer infectious disease in low income countries [5]. In most parts of Zimbabwe, *Plasmodium* *falciparum* is the most common and efficient malaria parasite that accounted for 99.7% of the estimated cases in 2018 [4] while *P.* *ovale* and *P.* *malariae* account for the remainder. The primary vector mosquito species responsible for most malaria transmission in Zimbabwe are *Anopheles* *arabiensis* and *Anopheles* *funestus* sensu stricto [6, 7].

In the past few years, malaria incidence and mortality have declined significantly across the globe [8, 9]. For instance, mortality rate decreased by 62% while malaria incidence decreased by 41% between 2000 and 2015 [10, 11]. The decline in malaria incidence and mortality is mainly attributed to malaria control interventions such as indoor residual spraying (IRS) and use of insecticide treated nets [10, 12]. Zimbabwe experienced a substantial decline in malaria cases of up to 81% from 2003 to 2015 across all age groups [13]. As a result of the substantial decline in malaria cases in Zimbabwe, the country adopted the global and regional agenda for malaria elimination by 2030 [14]. The target for the country is to reduce malaria incidence to 5/1000 by end of 2020 [15]. As malaria transmission continue to decline, prevention and control interventions will increasingly rely on accurate knowledge of the spatial distribution of high-risk geographic areas to support malaria elimination. This could be useful in optimal allocation of limited resources to ensure that areas with the highest malaria burden are given priority [16, 17]. Despite the declining burden of malaria, there still exist periodic outbreaks of malaria which exhibit spatial heterogeneity across different regions through time and space. Mapping malaria spatial heterogeneity is important to better understand transmission dynamics [18, 19].

The spatial heterogeneity in malaria transmission has resulted in malaria occurring in transmission clusters [19, 20]. The spatial heterogeneity in malaria is largely attributed to variation in environmental risk factors at the macro (e.g., temperature, precipitation) and the micro (e.g., local elevation, land use) spatial scales [21]. In this case, a malaria cluster is an area characterised by unusually high number of cases than expected within a population at a particular place at a given time [22]. As malaria occurrence exhibit spatial heterogeneity, strategies aimed at reducing or controlling the disease hinge upon objective and accurate characterisation of its clusters as a first step towards identifying areas with elevated malaria risk for prioritisation of interventions [18, 19]. Evidence from previous studies suggest that targeting malaria control interventions at high risk areas is cost effective and is bound to increase equity compared with undirected control [23]. Such targeted interventions ultimately reduce malaria mortality and morbidity [24]. Focussing malaria control in high risk areas is recommended by WHO in both elimination and post elimination settings [25].

Although previous studies assessed the spatial and temporal variation in malaria occurrence, most of these studies lacked the appropriate spatial scale that enables optimal planning at the national level [26–29]. In addition, most of the studies were either based on longitudinal cohort studies or limited in temporal duration [2, 30]. For example, some studies observed clustering of malaria cases at micro-geographic scale such as ward level in Gwanda district of Zimbabwe [31]. Similarly, it was also established that malaria exhibited spatio-temporal clusters at village level in China [29]. In addition, high malaria risk areas were identified in Hubei Province of China based on scan statistics [32]. In another study, it was found that malaria case distribution is characterised by spatial, temporal and spatiotemporal heterogeneity in unstable transmission areas in North-west Ethiopia [30]. Although these studies provide useful insights in understanding the spatial and temporal pattern of malaria, knowledge on the spatial and temporal pattern of malaria at a level where malaria interventions are commonly planned remain patchy. This is despite the fact that spatial analysis becomes much more meaningful when the spatial unit at which analysis is performed is representative of the expected epidemiological dynamics [21]. This will then mean the resulting national-level maps from such analysis will be justifiably utilized to prioritize high risk areas [21].

Despite the fact that effective malaria intervention warrants understanding of malaria heterogeneity at larger spatial scales for the purposes of resource allocation before focussing on microgeographic regions, studies at this scale remain largely limited. To fill this gap, this study utilised relatively long term malaria case data at district level, that is, the spatial epidemiological administrative unit at which malaria interventions and control are planned to determine not only persistent and stable clusters but emerging clusters as well [33]. The determination of spatial pattern of malaria at district level is also important in understanding possible interactions among neighbouring districts which is fundamental during malaria elimination.

To determine the spatial pattern of malaria clustering, it is important to adopt or even develop methods that can reliably and accurately detect malaria clusters in space and time. To date, several methods have been used to detect spatial and space-time clusters and these include, ClusterSeer [34], GeoSurveillance [35], kernel density [36], SaTScan [37] and Flex Scan [38]. The choice of a cluster detection technique can be guided by its sensitivity and specificity in addition to the power to detect clusters [39, 40]. In this study, SaTscan was applied since results from previous studies indicated that it has the highest overall sensitivity compared to other methods such as Local Indicators of Spatial Autocorrelation (LISA) and Getis [39] hence its ability to detect true clusters. Moreso, the technique maintains reasonably high power for detecting clusters compared to methods such as LISA which are influenced by neighbours [41]. Techniques such as Getis-Ord G_i_* statistic suffer from multiple testing which is inherently accounted for in SaTscan [42]. In this way, SaTscan combines exploratory and confirmatory capabilities which enable explicit statistical assessment of spatial pattern across the landscape [39].

In this study, the main objective was to test whether there is statistically significant spatial and space-time clustering of malaria at district level in Zimbabwe. The main hypothesis was that malaria tends to occur in clusters and that these clusters have both spatial and temporal characteristics.

## Methods

### Study area

**Fig. 1:**
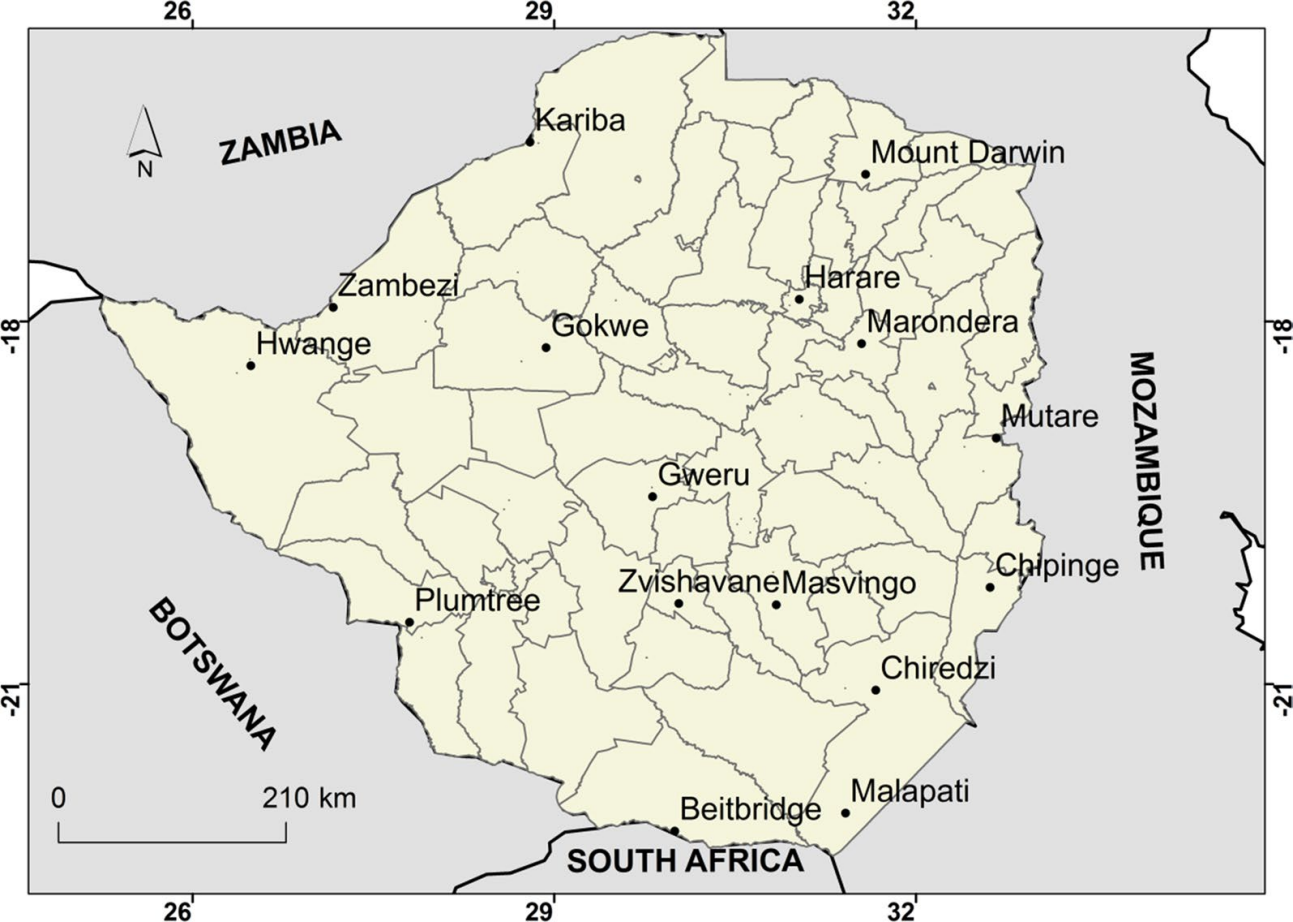
Location of the study area

The study was carried out in Zimbabwe located in southern Africa between latitudes 15° 30″ and 22° 30″ S and longitudes 25° 00″ and 33° 10″ E (Fig. 1). The altitude of the study area ranges from 300 to 2590 m above mean sea level. Mean annual rainfall ranges from below 400 mm in the southern and north-western parts to over 1000 mm in the eastern and central parts of the country. The country records its lowest minimum temperatures in June or July while the maximum temperatures occur in October. Mean monthly temperatures vary from 15 °C in July to 24 °C in November, while the mean annual temperature varies from 18 °C in the high-altitude areas to 23 °C in the low altitude areas. The study area is characterised by a subtropical climate with three recognisable seasons which are the hot wet season or summer stretching from mid-November to March; the cold dry season or winter stretching from April to July; and the hot dry season or spring from August to mid-November [43]. Zimbabwe experiences seasonal and spatial variation in malaria transmission that is related to the country’s climate especially rainfall pattern [13, 44]. The malaria peak transmission season in Zimbabwe is between February and April [13]. According WHO, there were close to 14 million people in the country, and four million were at risk of malaria with close to 1 million confirmed cases in 2018. The country is landlocked and shares the border with Mozambique, Botswana, Zambia and South Africa (Fig. 1).

## Data sources

### Malaria case data

The positive malaria cases recorded from 2011 to 2016 were obtained from geocoded health facilities in Zimbabwe and aggregated by month and year at district level*.* The country has over 1780 health facilities strategically placed within a 10-km radius in villages and urban areas [13]. The health facilities are ranked into central, provincial, and district hospitals, as well as clinics/rural health centres (RHCs). Data on malaria incidence at district level from 2011 to 2016 were collected based on the annual submission from health centres to DHIS 2. The data includes province, district, and centre name, date of diagnosis, age and gender. The data is for cases based on diagnostics using either rapid diagnostic tests (RDTs) or microscopy reported in each year [13]. Zimbabwe, like most developing countries in sub-Saharan Africa, adopted the District Health Information System (DHIS2) in 2010 to harmonise health data management [45]. The malaria cases at district level were plotted using ArcGIS 10.3 (ArcGIS Desktop: Release 10. Redlands, CA, USA) [46] by month and year to assess the distribution of malaria cases in the country.

For the Ministry of Health and Child Care, two main sources are used to feed the national surveillance system with routine malaria data i.e., the Health Management Information System (HMIS) and the Rapid Disease Notification System (RDNS) [47]. The RDNS, weekly short message service (SMS) is used to report malaria cases for approximately 95% of the health facilities. In addition, the HMIS obtains its data from monthly aggregated malaria cases and deaths from all health facilities [47]. In most developing countries in sub-Saharan Africa including Zimbabwe, routine health information systems are weak and there are widespread concerns about the quality and utility of malaria data generated from these systems [48, 49]. Despite concerns about data quality, Zimbabwe has made great strides on this aspect through government initiatives and international support. To improve data quality, the government adopted the Global Technical Strategy for Malaria 2016–2030 which stresses the need for adequate investment in the management and use of data from routine health information systems to support programme planning, implementation and evaluation [50].

### Population data

Population data used in this study were obtained from the Zimbabwe National Statistical Agency (ZimStat) based on the 2012 National population census [51]. The 2010 and 2011 population data was based on a 1.1% projected growth rate from the 2002 National census [52] while the 2012 data was based on the 2012 National Census. The population for intercensal years for example 2013 to 2015 were determined using the projected annual growth rate of 1.2% based on the 2012 national census [53]. The population of intercensal years is based on projected growth rates because the country conducts a population census after every 10 years.

### Statistical data analysis

#### Testing for spatial autocorrelation

Moran’s *I* [54], a global autocorrelation statistic was used to detect spatial pattern of malaria in the country. Using this technique, significant positive spatial autocorrelation of malaria cases imply that the distribution of malaria cases is more spatially aggregated than a random underlying spatial process.

The Moran’s Index takes the form;

1 $$I = \frac{n}{{S_{o} }} \frac{{\mathop \sum \nolimits_{i = 1}^{n} \mathop \sum \nolimits_{j = 1}^{n} w_{i,j} z_{i} z_{j} }}{{\mathop \sum \nolimits_{i = 1}^{n} z_{i}^{2} }}$$

where Z*i* is the deviation of an attribute for feature *i* from its mean (x_i_ – $\bar{X}$), w_i,j_ is the spatial weight between feature i and j, n is equal to the total number of features and S_o_ is the aggregate of all spatial weights.

Moran’s index ranges from −1 to +1 with a score of zero indicating the null hypothesis of no clustering. A positive score indicates clustering of malaria cases while a negative value shows that neighbouring areas are characterised by dissimilar malaria cases [55]. To perform spatial autocorrelation, the Queen Contiguity method was applied to define a weight matrix specifying the spatial relationships among the districts of Zimbabwe. This method was adopted since malaria is not directionally restricted and the districts are highly irregular in shape and size [56]. The significance of Moran’s *I* was assessed by employing Monte Carlo randomization where a statistically significant (*Z* score > 1.96) indicated that neighbouring districts have similar malaria cases at county level.

#### Detecting malaria clusters using SaTScan

In this study, scan statistics [42] was applied in SaTScan v9.6 (https://www.satscan.org/) software to detect high cluster rate of malaria. In this case, spatial scan statistic, based on the discrete Poisson model, was applied to identify purely spatial clusters of malaria cases by year. On the other hand, the space-time scan statistic, based on Space-Time Poisson model was adopted to determine the presence of space-time clusters of malaria cases by month over the study period. Three datasets were prepared for use in SaTScan and these were: a case file representing annual malaria cases per each district (*n* = 59) from 2011 to 2016; a coordinate file representing geographic coordinates of the centroid of each district; and a population file representing the projected total population for each year from 2011 to 2016 for the respective district.

The program identified statistically significant retrospective clusters based on annual malaria cases aggregated per district in Zimbabwe from 2011 to 2016. SaTscan tests whether the number of malaria cases within any spatial window exceeds the number expected by a random process [57]. To achieve this, the centroid of each district was first determined and extracted in a GIS environment. The spatial join function in a GIS was then used to link the annual malaria cases for each year to the centroid of the districts.

Next, the annual malaria cases per district were converted to SaTscan format for use in the detection and analysis of clusters. For determining clusters, a cylindrical window with a circular geographic base centred on each district centroid and with height corresponding to time was applied [57]. The default value of 50% of the population at risk was adopted as recommended in literature [37, 58, 59]. Thus, clusters with statistical significance of *P* < 0.05 were classified as significant clusters. As previously mentioned, the space-time clusters of malaria with high rates were detected using the retrospective space-time analysis based on the discrete Poisson model. To do this, data was arranged at a monthly scale from 2011 to 2016 and hence the time aggregation length was set to one month in SaTscan software. The space-time scan statistic was defined by a cylindrical window with a circular geographic base and whose height corresponded to a time interval i.e., a month in this case. The space-time analysis was applied to detect the seasonal pattern in malaria in the country. This technique is more robust as it combines exploratory and confirmatory capabilities which enable explicit statistical assessment of spatial patterns across the landscape [39] compared to Getis-Ord G_i_*.To detect significant space-time clusters, 999 Monte Carlo replications were performed under the null hypothesis of random distribution of malaria cases [32]. In this case, statistical significance was tested using a Poisson generalized log likelihood ratio test based on Monte-Carlo inference [32, 60].

The relative risk was calculated by comparing the observed number of cases of malaria within each window to the expected number, using a Poisson model. The most likely cluster (hereinafter, primary cluster) was identified based on the maximum log likelihood ratio [61]. In addition, other clusters with statistically significant log likelihood values were defined as secondary clusters. The criterion of *no* *geographical* *overlap* was used to report secondary clusters.

#### Cluster frequency analysis

To understand the persistence or emergence of potential malaria hotspots cluster frequency analysis was performed in a GIS environment. Specifically, the cluster frequency analysis was performed through counting the number of times a district was detected as part of a cluster using overlay analysis. This procedure yielded the number of times a district coincided with detected clusters whether primary or secondary.

## Results

### Variation in monthly malaria incidence

**Fig. 2:**
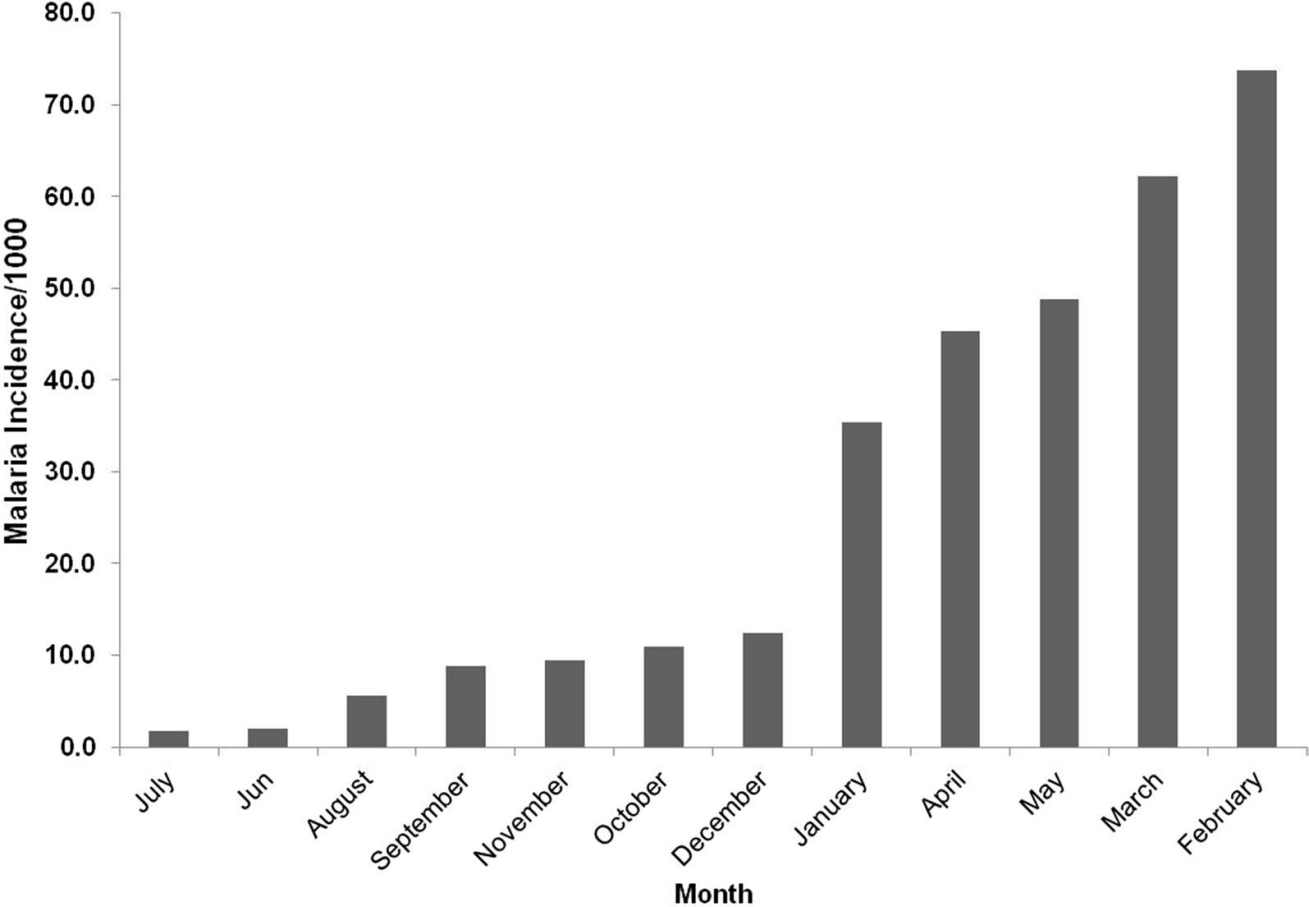
Average monthly malaria incidence from 2011 to 2016

Figure 2 illustrates the variation in average monthly malaria incidence from 2011 to 2016 in Zimbabwe. From 2011 to 2016, a total of 1 877 794 malaria cases were recorded throughout the country. It can be observed that malaria incidence start to increase from December and reach the highest peak in February. In contrast, the lowest incidence is recorded during the dry months such as August and cold month such as July.

### Annual incidence of malaria

**Fig. 3:**
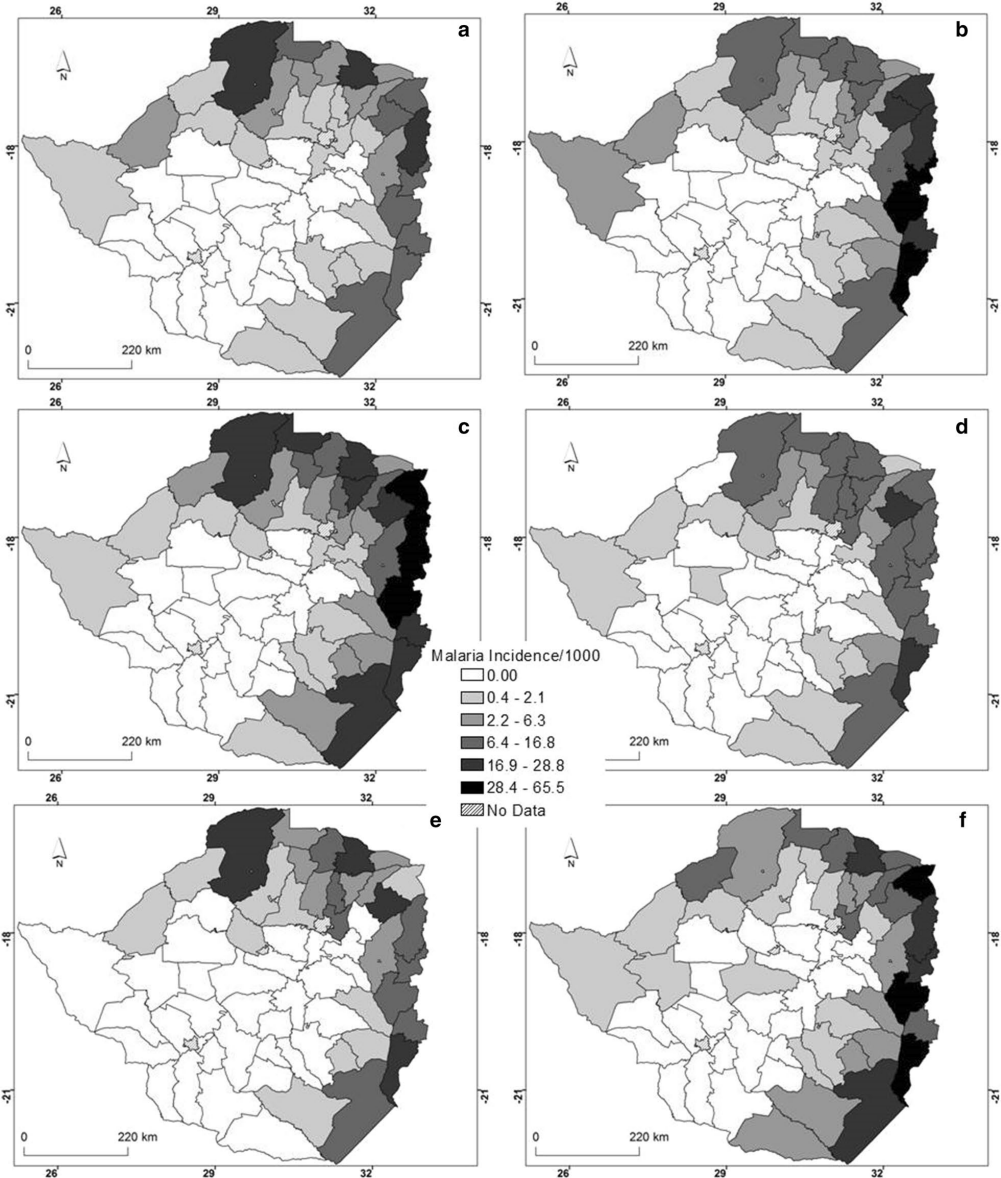
Spatial distribution of annual malaria incidence for **a** 2011, **b** 2012, **c** 2013, **d** 2014, **e** 2015 and **f** 2016

An analysis of annual malaria cases shows that over the 6 years, the northern, north-eastern, eastern and south-eastern districts of the country were characterised by high malaria incidence (Fig. 3). In contrast, the western, central and south western regions had low malaria incidence during the same period. In fact, more districts in the eastern districts of the country experienced high malaria incidence in any other year compared with other districts where malaria occurs.

### Spatial autocorrelation of malaria cases

Spatial autocorrelation results based on the annual malaria cases showed that there was significant overall spatial autocorrelation in Zimbabwe across all the study years (Table 1). The results demonstrate that malaria cases highly cluster at country level for all the years under study.

**Table 1:** Spatial autocorrelation test on malaria cases from 2011 to 2016

Year	Moran’s index	*Z* score	*P*-value
2011	0.53	20.00	0.00
2012	0.56	21.45	0.00
2013	0.41	15.67	0.00
2014	0.45	17.27	0.00
2015	0.57	21.48	0.00
2016	0.25	112.09	0.00

### Spatial clusters of malaria from 2011 to 2016

**Fig. 4:**
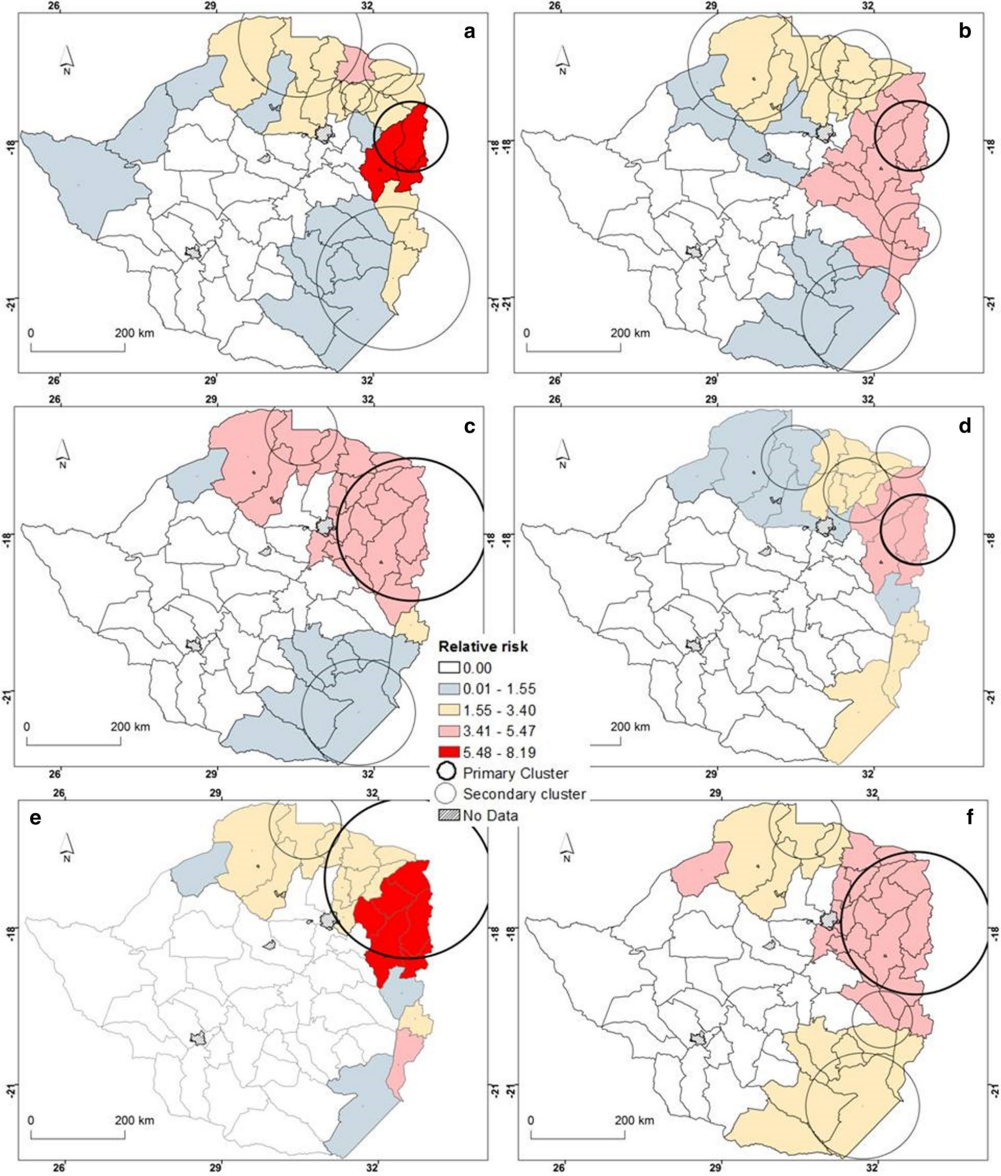
Spatial distribution of malaria clusters detected by purely spatial for **a** 2011, **b** 2012, **c** 2013, **d** 2014, **e** 2015 and **f** 2016. (*The* *primary* *cluster* *is* *illustrated* *by* *a* *darker* *outline*)

The results for the statistically significant (*P* < 0.05) primary and secondary spatial clusters as well as the corresponding relative risk for high rates of malaria occurrence identified by purely spatial scan statistic based on the discrete Poisson model are illustrated in Fig. 4. The results illustrate that there was significant spatial clustering of malaria cases in specific districts from the years 2011 to 2016 (Fig. 4, Table 2). Over the six years, primary clusters of malaria were concentrated in the eastern region of the country. The number of districts covered by the primary clusters increased from four in 2011 to 12 in 2016. In general, secondary clusters are characteristic of the eastern, northern, south-western and south-eastern regions of the country. However, the spatial location and size of these secondary clusters varied by year (Fig. 4).

**Table 2:** Significant malaria clusters detected using the purely spatial clustering

Year	Cluster type	Cluster areas (*n*)	Observed	Expected	RR	Radius(km)	LLR	*P*-value
	A	3	48 569	9569	6.38	74	44 198	0.00
	B	6	58 222	22 305	3.27	126	23 850	0.00
2011	B	2	13 937	4520	3.24	55	6 506	0.00
	B	7	47 530	36 405	1.40	152	1 937	0.00
	B	2	5414	5015	1.08	32	16	0.00
	A	3	92 324	17 148	6.94	74	89 397	0.00
	B	2	74 241	22 710	3.88	60	40 668	0.00
2012	B	5	44 030	25 085	1.86	73	6 386	0.00
	B	3	42 155	28 805	1.53	113	2 982	0.00
	B	5	34 236	31 941	1.08	122	89	0.00
	A	12	247 572	108 723	3.78	152	97 033	0.00
2013	B	2	41 339	10 382	4.28	74	27 258	0.00
	B	3	52 771	37 524	1.46	113	3 026	0.00
	A	3	52 868	12 087	5.28	74	40 965	0.00
	B	4	36 004	14 553	2.72	69	12 175	0.00
2014	B	2	16 088	5243	3.21	55	7 438	0.00
	B	3	17 509	14 423	1.23	69	329	0.00
	A	1	28 145	5788	5.47	114	23 439	0.00
2015	B	2	13 261	5885	2.34	74	3 533	0.00
	A	12	211 251	88 565	3.88	152	88 392	0.00
	B	2	79 698	23 800	3.92	60	44 713	0.00
2016	B	3	77 507	30 514	2.90	113	28 321	0.00
	B	2	31 355	12 709	2.59	74	10 118	0.00

*A* primary cluster, *B* secondary, *RR* relative risk; *LLR* log likelihood ratio

Further, results illustrate that the lowest number of malaria cases within a cluster was 5414 (2011) while the highest was 92 324 (2012). Across all the years under study, the most likely clusters had higher than expected malaria cases (Table 2).

### Frequency of cluster occurrence from 2011 to 2016

**Fig. 5:**
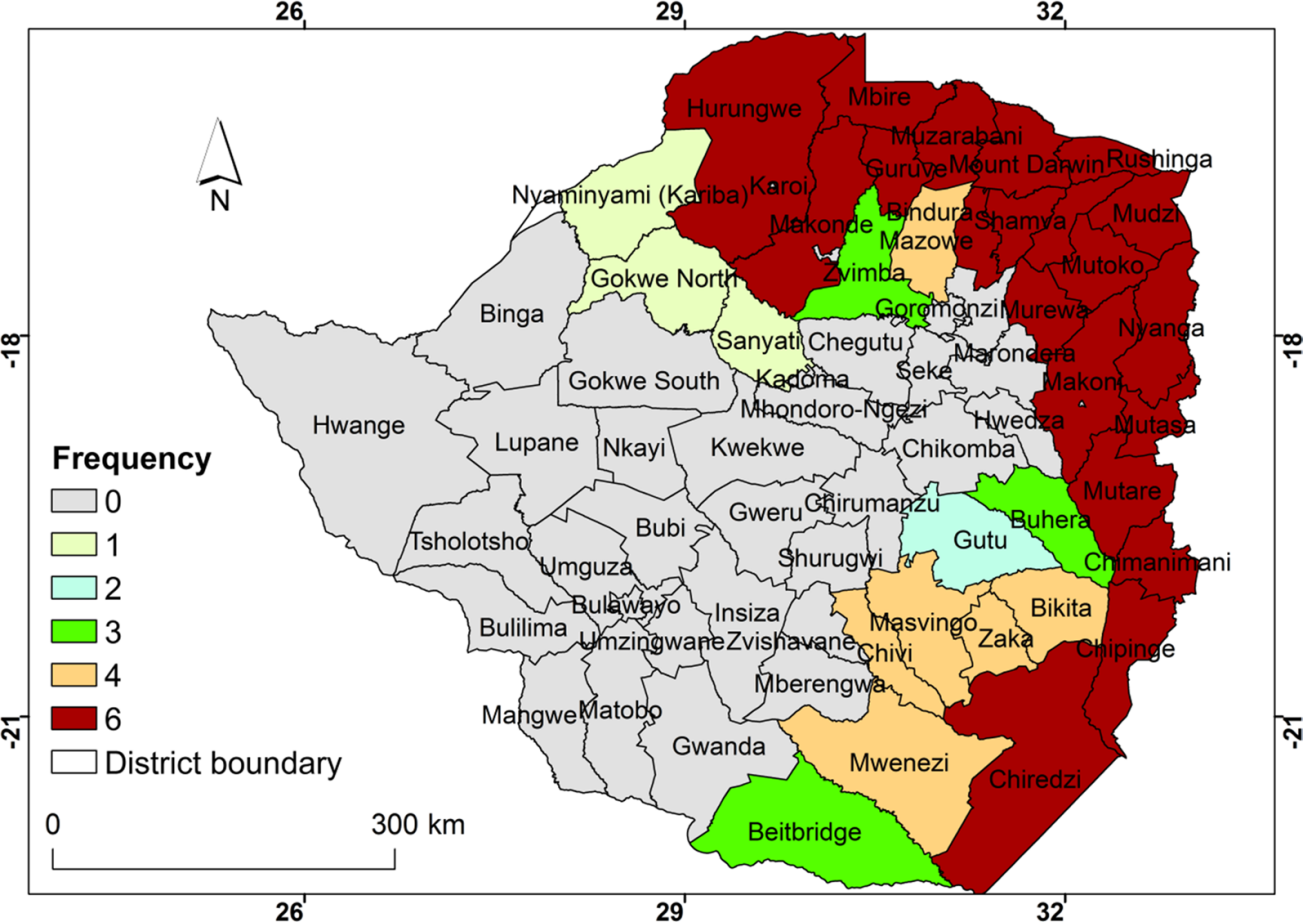
Frequency of cluster occurrence from 2011 to 2016

The frequency of occurrence of malaria clusters within districts based on scan statistics is illustrated in Fig. 5. It is observed that districts in the northern, north-eastern, eastern and south-eastern regions of the country had the highest frequency of malaria clusters. Districts at the margins of high malaria cluster districts had low frequency of cluster occurrence. In contrast the central, western and south western districts had no malaria clusters during the period under consideration.

### Space-time clusters of malaria

**Fig. 6:**
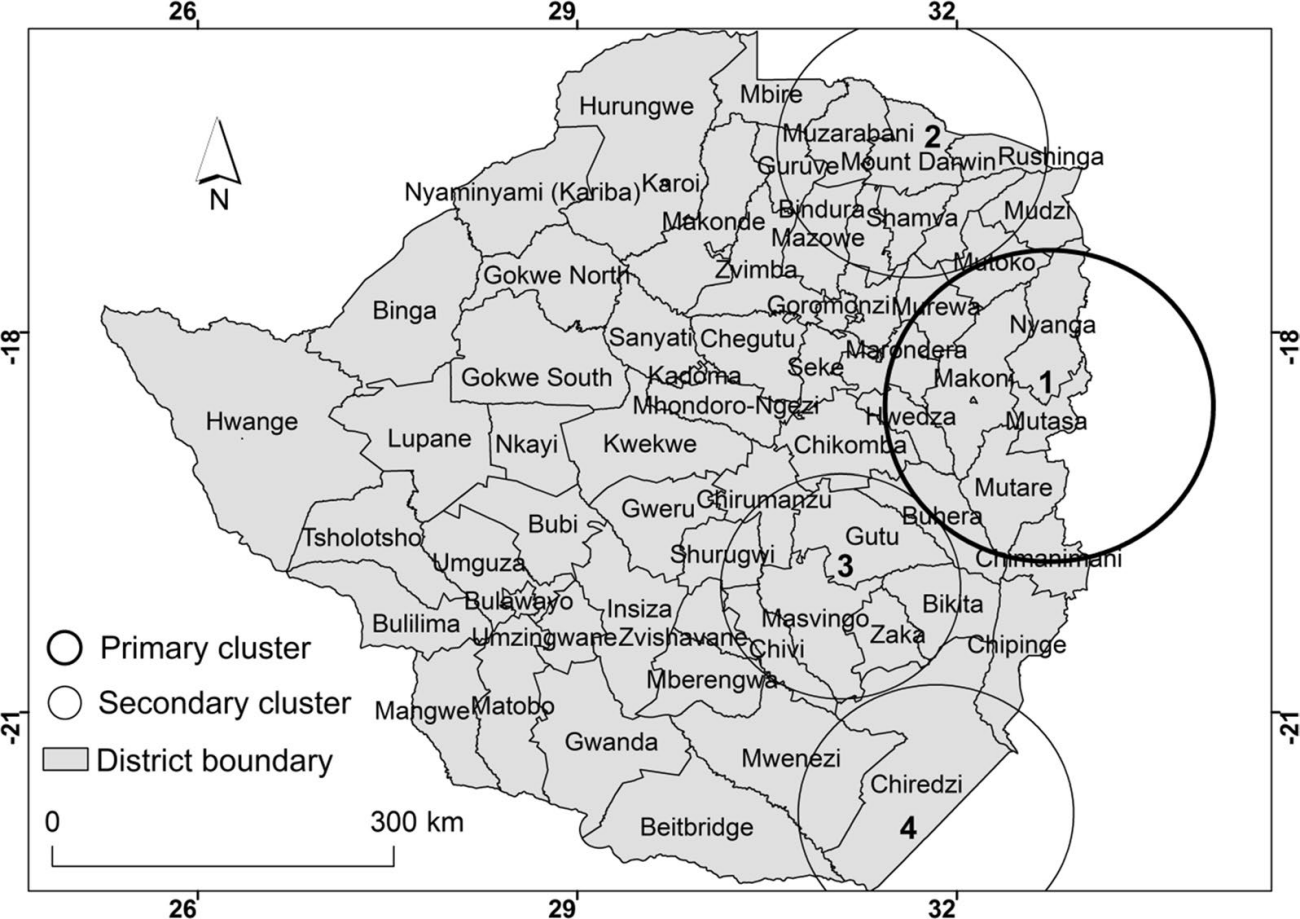
Spatial distribution of detected space-time clusters of malaria from 2011 to 2016

Results of space-time Poisson model show four spatial–temporal malaria clusters that were detected from 2011 to 2016 (Fig. 6, Table 3). The four statistically significant spatio-temporal clusters consisted of one primary cluster and three secondary clusters. The primary cluster was located in the north eastern region and covers eight administrative districts (Fig. 6).

**Table 3:** Spatial–temporal high risk clusters of malaria cases detected using space-time Poisson model from 2011 to 2017

Cluster #	# of Location	Start date	End date	LLR	*P* value	RR	Radius
*1	8	01/12/2012	31/05/2014	331 605	0.001	6.10	137
2	9	01/03/2014	31/05/2014	148 263	0.001	10.89	114
3	6	01/01/2016	31/05/2016	83 074	0.001	7.74	113
4	3	01/04/2014	31/05/2014	313	0.001	1.38	99

*Primary cluster

*RR* relative risk, *LLR* Log likelihood ratio

The primary cluster was detected from the 1st of December 2012 to the 31st of May 2014. The primary cluster persisted in this region for three seasons and covered eight districts (Table 3).

## Discussion

In this study, Geographic Information System coupled with a spatial scan statistical method were successfully applied to explore spatial and temporal patterns of malaria clusters between 2011 and 2016 at district level in Zimbabwe. The results of this study showed significant global spatial autocorrelation of malaria cases from 2011 to 2016 which indicates that the spatial distribution of malaria followed a clustered pattern. The results confirm findings from previous studies that observed the tendency of malaria to cluster in particular geographic regions mostly derived by the spatial heterogeneity in the factors that drive transmission of the disease [20, 31]. Based on previous studies, the spatial heterogeneity in malaria is largely attributed to variation in environmental risk factors at the macro (e.g., temperature, precipitation) and the micro (e.g., local elevation, land use) spatial scales [21]. From the observed detected pattern, the variation in malaria cases coincide with the distribution of the preferred habitat of the *Anopheles* mosquitoes which are the main vectors that transmit *P.* *falciparum* parasite [21]. Thus, our study further provides evidence of spatial heterogeneity in the occurrence of malaria in the affected regions.

Results of purely spatial analysis based on the poisson model showed that primary and secondary clusters of malaria persisted in the northern, north-eastern, eastern and south-eastern districts of Zimbabwe. The results show that consistently over the study period, malaria clusters occur in different sizes and at different locations. This is important in identifying stable cluster areas which persist in areas of high malaria burden [20]. The results support the hypothesis that malaria cases tend to significantly cluster within certain geographic units albeit with observable shifts over time. This may indicate that the occurrence of malaria in Zimbabwe is characterised by spatial heterogeneity as high-risk areas still exist particularly in the north north-east, east and south eastern districts of the country [31]. The high risk areas detected in this study are consistent with malaria hotspots detected through Getis G_i_* statistic analysis and were closely related to high vector habitat suitability [2]. In addition, the high risk areas coincide with the high and perennial malaria risk zones delineated through malaria risk stratification in Zimbabwe [62]. As the country moves towards malaria elimination [13], there is need to prioritise control efforts by focussing on high risk areas as these are possible reservoirs of malaria transmission [63]. The detection of statistically significant malaria clusters is a critical step towards spatial targeting and selection of appropriate population level interventions as these clusters are potential reservoirs for future infection [20, 27]. Through the detection of clusters, affected countries can shift from malaria control to malaria elimination which is one of the key goals of the WHO Global Technical Strategy for Malaria 2016–2030 [14]. The targeting of high risk areas for malaria control aligns with the United Nations Sustainable Development Goal (SDG) number three which is seeks to promote good health and well-being through scaling up of malaria interventions [4, 64].

Results of space-time analysis showed that malaria clusters tend to occur in particular months e.g. December to May. The fact that most clusters were detected during this period is not surprising as the country receives most of its rainfall between December and March which dissipates towards April and May [13]. The high amount of rainfall coupled with relatively high temperatures during this period provides optimal conditions for mosquito breeding and subsequent malaria transmission [13]. Previous studies have shown that this period coincides with the malaria epidemic season in Zimbabwe and offers favourable climatic conditions in high risk months for malaria transmission [7, 13, 44]. When combined with location specific information on malaria clustering, results of space-time clustering further points to the importance of incorporating these two aspects in order to fully understand malaria transmission dynamics. Such insights would not have been generated had the study only adopted either purely spatial or purely temporal approach in modelling clustering of malaria. The results of space-time analysis can then be utilised to plan timing of control interventions by targeting those months where clusters are common. This would require deviation from the usual practice where indoor residual spraying is done well before the malaria season.

What makes this study different from other previous studies is that, unlike previous studies, this study used malaria case data for a relatively long period (six years) which provides important insights in the persistence of clusters (stable clusters) in certain geographic regions. In addition, this study integrated space and time in one analytical framework which provides new insights into the evolution of malaria not only in the spatial but also in the temporal domain. This study utilised one of the most robust methods of cluster detection to understand the pattern of malaria clusters unlike previous studies which have mostly utilised hotspot analysis techniques such the Getis G_i_* statistic [2]. The technique used in this study has both high specificity and sensitivity hence provides a balance in terms of committing at type 1 or type 2 errors. Furthermore, the high risk areas identified in this study may serve as important starting points for future disease surveillance in resource limited environments such as Zimbabwe. Apart from providing disease surveillance targets, such high risk areas could be prioritised during resource allocation to achieve effective disease control. However, further research should be focussed in these areas to fully understand disease etiology and local factors that support elevated malaria risk.

Data quality related to use of retrospective data may have affected the results of this study. Most developing countries are characterised by incomplete reporting of routine data, non-reporting, missing data and poor data aggregation frameworks [65]. Nonetheless, malaria case-management and data quality have greatly improved particularly parasitological testing as well as the adoption of electronic databases such as DHIS2. Although routine data from health facilities is known to underestimate malaria burden due to the above mentioned factors, the data is still useful in understanding the spatial distribution of malaria in endemic regions [66, 67]. Thus, the results of this study provide an important basis for planning and implementation of malaria control strategies.

Although spatial and spatio-temporal clusters of malaria were successfully detected using data from 2011 to 2016, one limitation is that the malaria cases used in this study did not differentiate local and imported cases. It is important to differentiate local and imported malaria cases particularly given the observation that most of the high rates of malaria clusters tend to be concentrated along borderline areas [68, 69]. The challenge is that a greater part of the borders of the country are porous making it difficult to monitor movement. For example, to the east Zimbabwe shares a 730 km border with Mozambique which is also known to have high malaria cases while to the south-east the country borders with South Africa along the Limpopo valley (a malaria endemic region) [70–72] and to the North it borders with Zambia [73, 74]. Imported cases could have influenced the size and location of high rates of malaria clusters detected in this study. Migration related malaria remains a major problem for Zimbabwe especially in the eastern parts of the country. The occurrence of malaria due to migration could be as a result of locals travelling to neighbouring Mozambique during the day and contracting malaria which is then reported in eastern districts. Additionally, populations from neighbouring country may access treatment in Zimbabwe’s eastern districts where the treatment is free to patients. Usually these cases are not reported in the DHIS2 database despite receiving treatment. The use of genomic surveillance may address the first challenge of locals contracting malaria from the neighbouring country. Introducing a data point recording non-resident malaria patients would allow an accurate characterisation of the burden of malaria in the eastern border districts. There is therefore need for national data collection systems to incorporate imported cases in their systems. To achieve this, there is need for closer collaboration with neighbouring countries.

The information generated in this study could be important in strengthening cross border collaboration given that the country has joined other Southern African countries to achieve malaria elimination [13, 73]. This will be achieved through alliances such as Elimination8 (E8) comprising Angola, Botswana, Mozambique, Namibia, South Africa Swaziland, Zambia and Zimbabwe. Closer collaboration in malaria elimination could be achieved through the ZAMZIM (Zambia and Zimbabwe), and the MOZAZI (Mozambique, Zambia and Zimbabwe), and the MOZIZA (Mozambique, Zimbabwe and South Africa) initiatives [3, 13, 73].

Nevertheless, insights generated in this study are useful in guiding further research on tightening cross border migration to malaria transmission and strengthening collaboration among neighbouring countries in the control of malaria. This is because without collaboration, malaria elimination is bound to fail as malaria occurrence due the influence of imported cases. Another potential limitation of the study is that although Kulldorff’s scan statistic has been successfully used to detect circular clusters, it does not have the same success rate when detecting irregular clusters [75]. Despite these potential limitations, the results of this study are still important and may be useful for planning disease surveillance, particularly in areas of limited resources by focussing on high risk areas.

## Conclusions

This study explored whether there is spatial heterogeneity in the distribution of malaria, one of the diseases of global public health concern. This was achieved through the detection of spatial and space-time clusters using scan statistics. The results indicated that high risk areas for malaria are concentrated in the northern, eastern, and south-eastern part of the country. The results of this study could be used to design malaria control strategies aimed at reducing malaria incidence in high risk areas particularly those along border areas. In addition, the results could be used to guide optimal resource allocation by giving priority to the regions in greatest need. The results of this study highlight the spatial heterogeneity in malaria occurrence with several high-risk areas detected across the country. Based on this retrospective study, significant attention need to be directed to high risk areas as these may act as reservoirs for the current and future malaria occurrence. The study is helpful in prioritizing resource allocation in high-risk areas for effective disease control. Although results are based on historical data, they are useful in tracking progress the country has made in reducing malaria incidence. In addition, the results can be used as a baseline to evaluate the impacts of malaria programmes implemented during this period which is important in informing current and future control strategies.

## Data Availability

The statistical methods presented in this manuscript were implemented in a freely downloadable software SaTScan version 9.4.2 available at (https://www.satscan.org/). The data used and/or analysed during the current study are available from the corresponding author on reasonable request.

## Abbreviations

DHIS: District Health Information System
GIS: Geographic Information System
IRS: Indoor residual spraying
MOZAZI: Mozambique, Zambia and Zimbabwe
MOZIZA: Mozambique, Zimbabwe and South Africa
SDG: Sustainable Development Goal
WHO: World Health Organisation
ZAMZIM: Zambia and Zimbabwe
ZIMSTAT: Zimbabwe National Statistics
